# Supplementary material for: Growth phase diets diminish histone acetyltransferase Gcn5 function and shorten lifespan of Drosophila males
Source: EMBO Rep. 2025 Jul 10;26(15):3856–88. doi: 10.1038/s44319-025-00503-8 (PMC12332192; doi:10.1038/s44319-025-00503-8)
Supplement: Supplementary file 1 — Appendix [file 44319_2025_503_MOESM1_ESM.pdf]

Growth phase diets diminish histone acetyltransferase Gcn5 function and shorten lifespan of *Drosophila* males

Table of Appendix Contents

Appendix Figure S1: Pages 2-3

Appendix Figure S2: Pages 4-5

Appendix Figure S3: Pages 6-8

Appendix Figure S4: Pages 9-10

Appendix Figure S5: Pages 11-12

Appendix Figure S6: Pages 13-14

References: Page 15

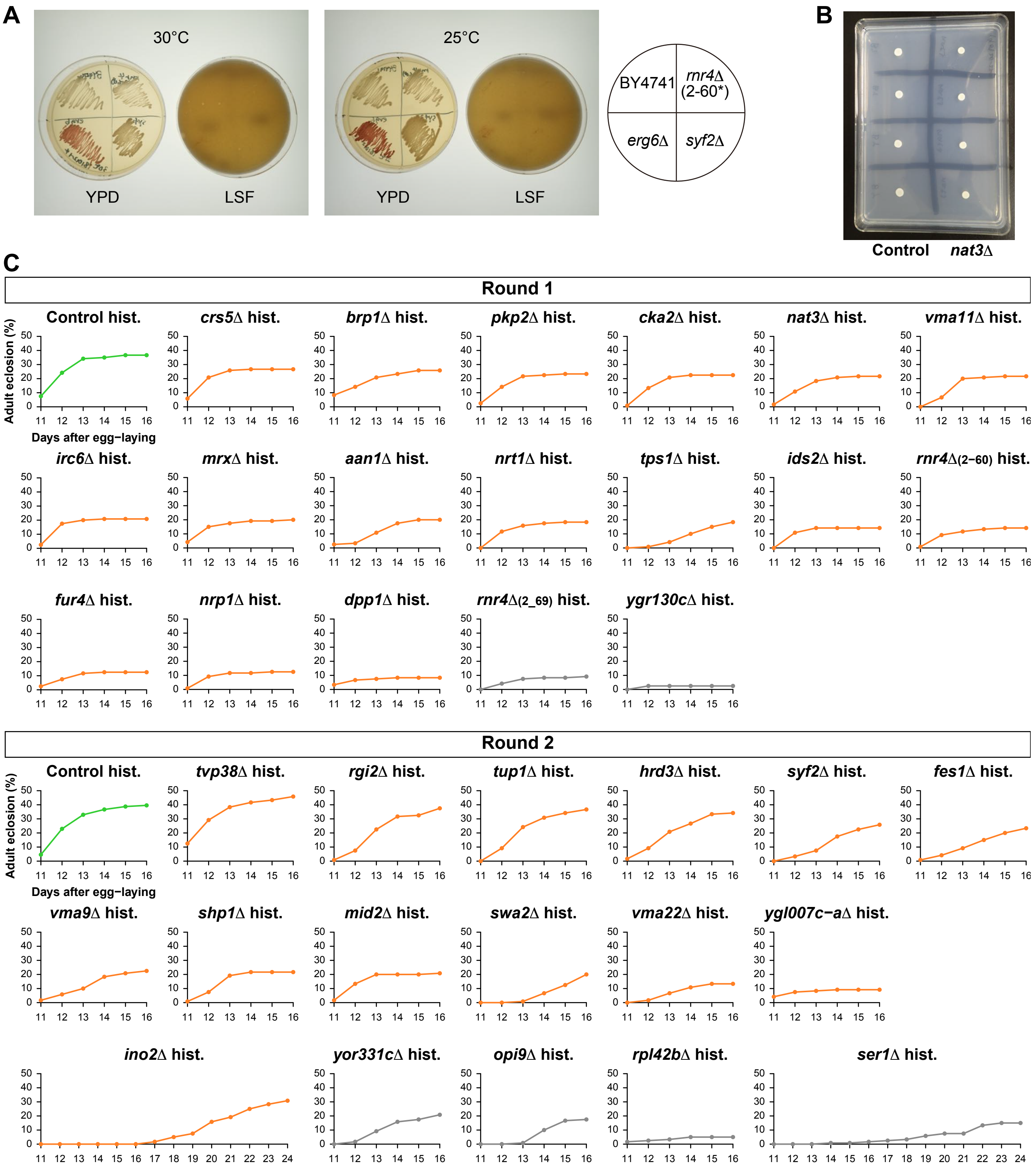
A
30°C
YPD
LSF
25°C
YPD
LSF
BY4741
rnr4Δ (2-60*)
erg6Δ
syf2Δ
B
Control
nat3Δ
C
Round 1
Control hist.
crs5Δ hist.
brp1Δ hist.
pkp2Δ hist.
cka2Δ hist.
nat3Δ hist.
vma11Δ hist.
Adult eclosion (%)
Days after egg−laying
irc6Δ hist.
mrxΔ hist.
aan1Δ hist.
nrt1Δ hist.
tps1Δ hist.
ids2Δ hist.
rnr4Δ(2−60) hist.
fur4Δ hist.
nrp1Δ hist.
dpp1Δ hist.
rnr4Δ(2_69) hist.
ygr130cΔ hist.
Round 2
Control hist.
tvp38Δ hist.
rgi2Δ hist.
tup1Δ hist.
hrd3Δ hist.
syf2Δ hist.
fes1Δ hist.
Adult eclosion (%)
Days after egg−laying
vma9Δ hist.
shp1Δ hist.
mid2Δ hist.
swa2Δ hist.
vma22Δ hist.
ygl007c−aΔ hist.
ino2Δ hist.
yor331cΔ hist.
opi9Δ hist.
rpl42bΔ hist.
ser1Δ hist.

Appendix Figure S1. Cell growth of the yeast mutants and effects of yeast mutant diets on percentage and timing of adult eclosion

(A) Yeast *S. cerevisiae* cannot grow on our standard food (laboratory standard food, LSF), which contains anti-fungal reagents. In contrast to growth promotion on the rich medium for yeast (YPD), none of the four strains tested formed colonies on LSF: the control yeast, BY4741, in the top left quadrant, followed clockwise by *rnr4Δ (2-60*)*, *syf2Δ* and *erg6Δ*. This was the case with both incubation conditions, at 30°C for 2 days (the temperature used for culturing yeast) and at 25°C for 7 days (the temperature used for rearing *D. melanogaster*). This result indicates that in our live yeast-fly assay, yeast cells from the larval diet have a minimal effect on lifespan even if they happened to be carried over to LSF with eclosed adults (Figure 1A). (B) Suspensions of the control yeast and *nat3Δ* were prepared exactly as for the live yeast-fly assay (see details in Methods) and 5 μl of each suspension was spotted on a mSCM plate. The plate was cultured at 30°C for 2 days. There was no obvious difference in cell growth between the two yeast strains. (C) As shown in Figure 1C, the 46 yeast gene KO diets altered the timing and/or the rate of pupariation compared to the control yeast. Out of those diets, 11 failed to support the production of pupae, and these 11 were excluded from the subsequent assay. Three of the 11 strains were *erg2Δ*, *erg3Δ* and *erg24Δ*, which are defective in sterol biosynthesis. We divided the remaining 35 yeast strains into two groups (Round 1 and 2) and fed *Canton-Special* larvae on each strain. In each plot, the vertical axis indicates the adult eclosion percentage that was calculated from the daily number of eclosed male and female adults out of 120 germ-free embryos (30 embryos per replicate, total 4 replicates). A total of 29 yeast strains (orange curves) allowed larvae to produce sufficient numbers of adults for the lifespan assay. See yeast strains of individual categories in Dataset EV1.

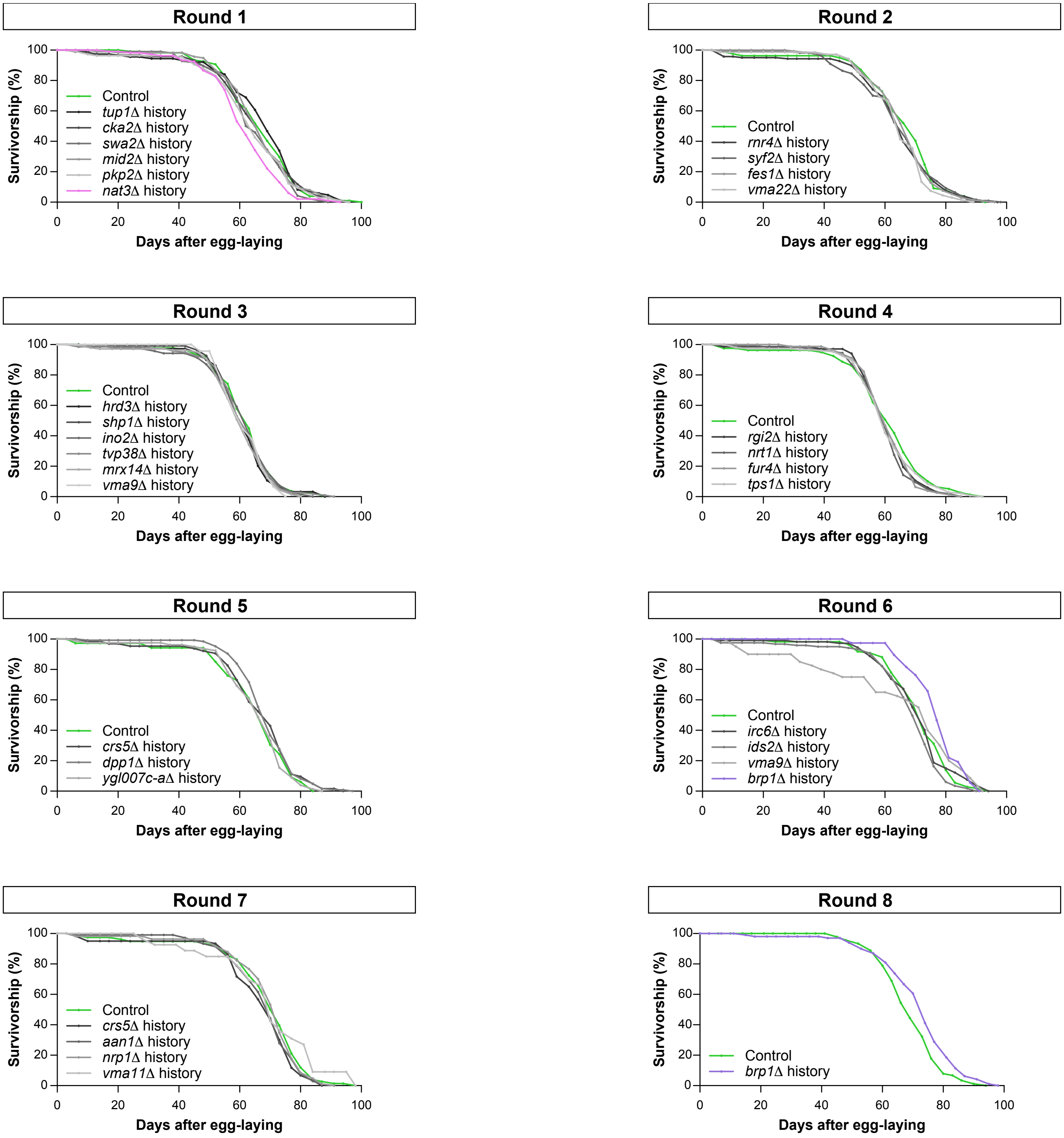
Round 1
Survivorship (%)
Control
tup1Δ history
cka2Δ history
swa2Δ history
mid2Δ history
pkp2Δ history
nat3Δ history
Days after egg-laying
Round 2
Survivorship (%)
Control
rnr4Δ history
syf2Δ history
fes1Δ history
vma22Δ history
Days after egg-laying
Round 3
Survivorship (%)
Control
hrd3Δ history
shp1Δ history
ino2Δ history
tvp38Δ history
mrx14Δ history
vma9Δ history
Days after egg-laying
Round 4
Survivorship (%)
Control
rgi2Δ history
nrt1Δ history
fur4Δ history
tps1Δ history
Days after egg-laying
Round 5
Survivorship (%)
Control
crs5Δ history
dpp1Δ history
ygl007c-aΔ history
Days after egg-laying
Round 6
Survivorship (%)
Control
irc6Δ history
ids2Δ history
vma9Δ history
brp1Δ history
Days after egg-laying
Round 7
Survivorship (%)
Control
crs5Δ history
aan1Δ history
nrp1Δ history
vma11Δ history
Days after egg-laying
Round 8
Survivorship (%)
Control
brp1Δ history
Days after egg-laying

Appendix Figure S2. Lifespans of male adults that had been subjected to 29 different yeast strain diets in their larval stages

white Dahomey larvae were fed on each of the 29 yeast strains, which had been selected as described in Appendix Figure S1, or on the control yeast. The emerged male adults were aged on the standard laboratory food and their lifespan was measured. As a result, larval diets of 2 yeast strains, *nat3Δ* (pink in Round 1) and *brp1Δ* (purple in Rounds 6 and 8), altered lifespan compared to the control yeast diet. See more details in Dataset EV1 and EV2. Growth of *ino2Δ* yeast on mSCM was exceedingly poor compared to control yeast. Consequently, *ino2Δ*-history adults, which had experienced such a poor nutrition condition, started to emerge 6 days after control adults (Round 2 in Appendix Figure S1). Nevertheless, the *ino2Δ* history did not affect male lifespan (Round 3). Round 8 experiment was performed simultaneously with the experiment of Figure EV1D. The exact *P* values, sample sizes and statistical tests employed are listed in Dataset EV12.

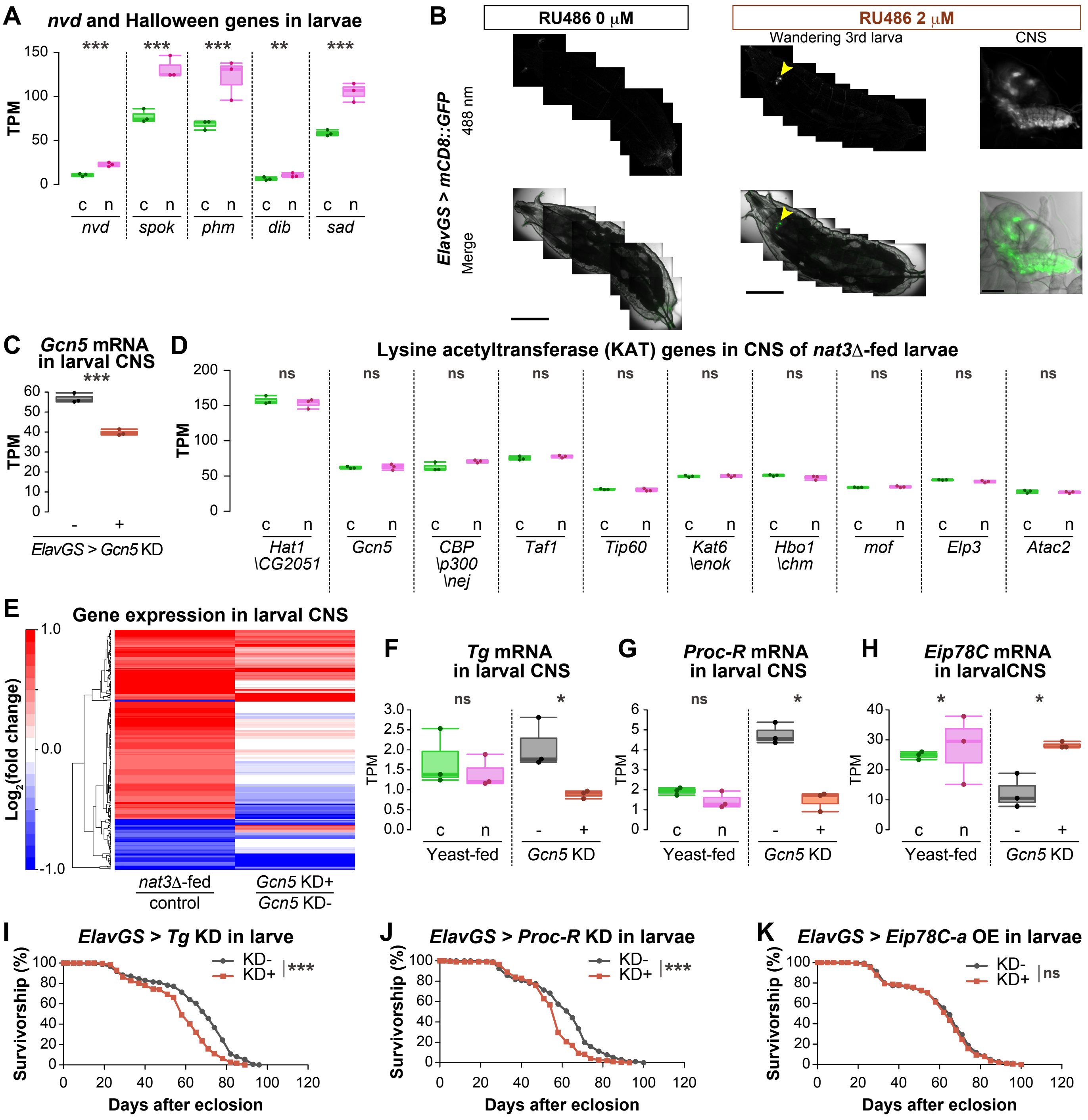
A
nvd and Halloween genes in larvae
TPM
c n
nvd
spok
phm
dib
sad
B
RU486 0 μM
RU486 2 μM
Wandering 3rd larva
CNS
ElavGS > mCD8::GFP
488 nm
Merge
C
Gcn5 mRNA in larval CNS
ElavGS > Gcn5 KD
D
Lysine acetyltransferase (KAT) genes in CNS of nat3Δ-fed larvae
Hat1 \CG2051
Gcn5
CBP \p300 \nej
Taf1
Tip60
Kat6 \enok
Hbo1 \chm
mof
Elp3
Atac2
E
Gene expression in larval CNS
Log2(fold change)
nat3Δ-fed control
Gcn5 KD+ Gcn5 KD-
F
Tg mRNA in larval CNS
G
Proc-R mRNA in larval CNS
H
Eip78C mRNA in larvalCNS
Yeast-fed
Gcn5 KD
I
ElavGS > Tg KD in larve
J
ElavGS > Proc-R KD in larvae
K
ElavGS > Eip78C-a OE in larvae
KD-
KD+
Survivorship (%)
Days after eclosion

Appendix Figure S3. Other characterizations of RNA-seq of the *nat3Δ*-fed male larvae and the neuron-specific *Gcn5* knockdown

(A) Expression values (transcripts per million; TPM) of *neverland* (*nvd*) and 4 Halloween genes, *spookier* (*spok*), *phantom* (*phm*), *disembodied* (*dib*), and *shadow* (*sad*), in our larval whole-body RNA-seq data. All these genes were significantly upregulated in *nat3Δ*-fed larvae (pink, “n”) compared to the control (green, “c”). They belong to the KEGG “insect hormone biosynthesis” pathway (Appendix Figure S4B) and encode ecdysteroidogenic enzymes that catalyze reactions from sterol to 20-hydroxyecdysone and/or other ecdysteroids (Niwa & Niwa, 2016). It has been reported that expression of Halloween genes, including the above four, is regulated by the *Drosophila* ATAC complex that contains Gcn5 and Ada2a as subunits (Pankotai et al., 2010), which prompted us to pursue the relationship between the *nat3Δ* diet and Gcn5 function. It should be noted that the Halloween genes are downregulated in the *Ada2a* mutant (Pankotai et al., 2010) in contrast to the upregulation by the *nat3Δ* diet. (B) Representative images of wandering 3rd-instar larvae that contain the *ElavGS* and *UAS-mCD8::GFP* constructs. The left and right panels show whole-body images of male wandering 3rd larvae that developed on a standard food with no chemical (RU486, 0 μM) and with the chemical (RU486, 2 μM), respectively. The top images are those of a 488 nm laser channel and the bottom ones are images merged with a transmitted light. On the far righthand side are enlarged images of dissected central nervous system (CNS). mCD8::GFP expression in CNS is marked with yellow arrow heads. The scale bar represents 1,000 μm for the whole-body images and 100 μm for the dissected CNS. (C) Quantity of *Gcn5* transcripts shown by RNA-seq of larval CNS. “-” (gray) indicates no induction of *Gcn5* short hairpin RNA, whereas “+” (orange) indicates the induction in larval neurons. (D) Expression values (TPM) of lysine acetyltransferase (KAT) genes (Feller et al., 2015) in our larval CNS RNA-seq data. Given that Gcn5 function in larval neurons is critical for adult lifespan, we assumed that the *nat3Δ* diet reduces the Gcn5 function in larval neurons, much as we observed in the whole-larval samples (Figure 2). Furthermore, we hypothesized that the *nat3Δ* diet-induced reduction in the Gcn5 function led to abnormal expression patterns of (a) group(s) of genes in larval neurons, which may be an indirect cause of the lifespan shortening. To search for such hypothetical genes, we conducted RNA-seq of the larval CNS under the dietary and knockdown conditions (Dataset EV7). We first confirmed that the larval *nat3Δ* diet did not affect the *Gcn5* expression and other KAT genes in the larval CNS as shown in panel D. (E) A heat map of gene expression levels in the CNS whose $\log_2$ fold changes between control and *nat3Δ*-fed larvae [$\log_2$(*nat3Δ*-fed|control)] were smaller than -0.5 (blue) or larger than 0.5 (red). The right column “*Gcn5* KD+|*Gcn5* KD-” shows whether each of these genes tended to be up or down in the *Gcn5* KD larvae (KD+) compared to KD-. (F-H) Gene expression values (TPM) of *Transglutaminase* (*Tg*, panel F), *Proctolin receptor* (*Proc-R*, panel G) and *Ecdysone-induced protein 78C* (*Eip78C*, panel H). These 3 genes were considered as key for lifespan shortening, whose expression changes between the conditions were not perfectly significant but either lower or higher both on the *nat3Δ* diet and under the neuronal *Gcn5* KD. Tg catalyzes crosslinking of proteins by forming epsilon-gamma glutamyl lysine isopeptide bonds, and *Tg* mutant adults show age-dependent neurodegeneration in the brain and die earlier than the control adults (Kounatidis et al., 2017). Proc-R is a receptor for a neuropeptide hormone proctolin, and Proc-R-expressing neurons in the larval brain include neurosecretory cells (Johnson et al., 2003). Eip78C is a nuclear hormone receptor that is most similar to human Rev-Erb, a member of the same superfamily as PPARs (Thummel, 1995). (I-K) Here are survival curves of adults with neuronal KD histories of *Tg* (I) and *Proc-R* (J) or neuronal overexpression history

of *Eip78C isoform A* (K). Either gene KD selectively in larval neurons significantly shortened adult lifespan, whereas *Eip78C* overexpression in larval neurons did not affect the adult lifespan. To summarize our small-scale experiment, we found at least two candidate genes whose perturbed expression in larval neurons leads to long-term effects on the adult lifespan. The data of I-K were obtained in a set of experiments. Boxplots are depicted as in “Statistical analysis” in Methods. $^{*}P < 0.05$, $^{**}P < 0.01$, $^{***}P < 0.001$. The exact P values, sample sizes and statistical tests employed are listed in Dataset EV12.

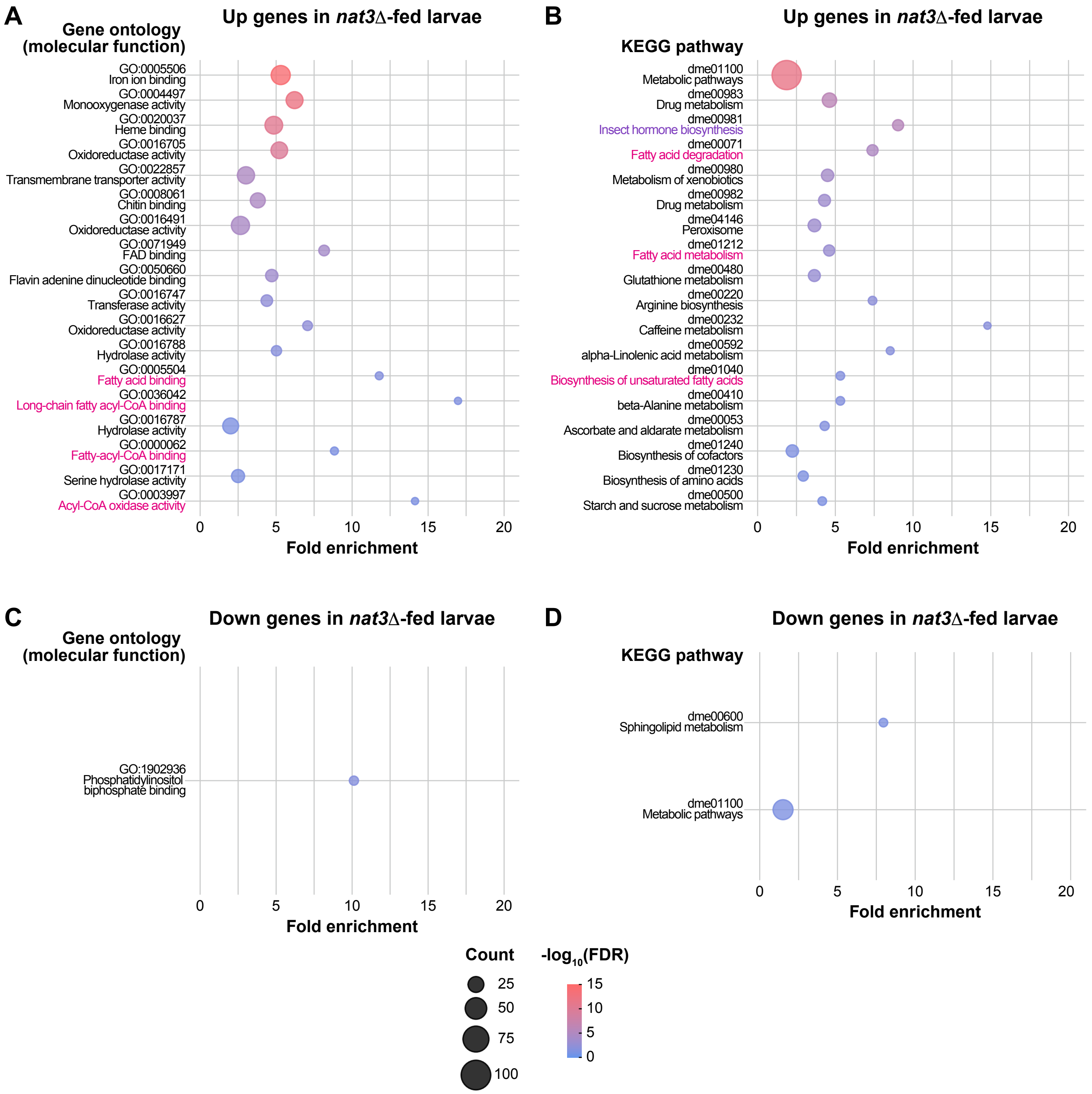
A
Up genes in nat3Δ-fed larvae
Gene ontology (molecular function)
GO:0005506 Iron ion binding
GO:0004497 Monooxygenase activity
GO:0020037 Heme binding
GO:0016705 Oxidoreductase activity
GO:0022857 Transmembrane transporter activity
GO:0008061 Chitin binding
GO:0016491 Oxidoreductase activity
GO:0071949 FAD binding
GO:0050660 Flavin adenine dinucleotide binding
GO:0016747 Transferase activity
GO:0016627 Oxidoreductase activity
GO:0016788 Hydrolase activity
GO:0005504 Fatty acid binding
GO:0036042 Long-chain fatty acyl-CoA binding
GO:0016787 Hydrolase activity
GO:0000062 Fatty-acyl-CoA binding
GO:0017171 Serine hydrolase activity
GO:0003997 Acyl-CoA oxidase activity
0 5 10 15 20
Fold enrichment
B
Up genes in nat3Δ-fed larvae
KEGG pathway
dme01100 Metabolic pathways
dme00983 Drug metabolism
dme00981 Insect hormone biosynthesis
dme00071 Fatty acid degradation
dme00980 Metabolism of xenobiotics
dme00982 Drug metabolism
dme04146 Peroxisome
dme01212 Fatty acid metabolism
dme00480 Glutathione metabolism
dme00220 Arginine biosynthesis
dme00232 Caffeine metabolism
dme00592 alpha-Linolenic acid metabolism
dme01040 Biosynthesis of unsaturated fatty acids
dme00410 beta-Alanine metabolism
dme00053 Ascorbate and aldarate metabolism
dme01240 Biosynthesis of cofactors
dme01230 Biosynthesis of amino acids
dme00500 Starch and sucrose metabolism
0 5 10 15 20
Fold enrichment
C
Down genes in nat3Δ-fed larvae
Gene ontology (molecular function)
GO:1902936 Phosphatidylinositol biphosphate binding
0 5 10 15 20
Fold enrichment
D
Down genes in nat3Δ-fed larvae
KEGG pathway
dme00600 Sphingolipid metabolism
dme01100 Metabolic pathways
0 5 10 15 20
Fold enrichment
Count
25
50
75
100
-log10(FDR)
15
10
5
0

Appendix Figure S4. Molecular function and pathways of differentially expressed genes in the *nat3Δ*-fed male larvae

(A and B) Plots showing the result of Gene Ontology (GO) molecular function term (A) or KEGG Pathway (B) enrichment analysis (DAVID Functional Annotation Chart) of the upregulated (Up) genes in the *nat3Δ*-fed larvae. (C and D) Those of downregulated (Down) genes in the *nat3Δ*-fed larvae. Terms/pathways with *FDR* smaller than 0.05 in an individual analysis are listed. The circle size indicates the number of genes in each category. The circle color represents the transformed *FDR* value. GO terms related to "fatty acid" (magenta) were enriched. The pathway name "Insect hormone biosynthesis" is colored purple.

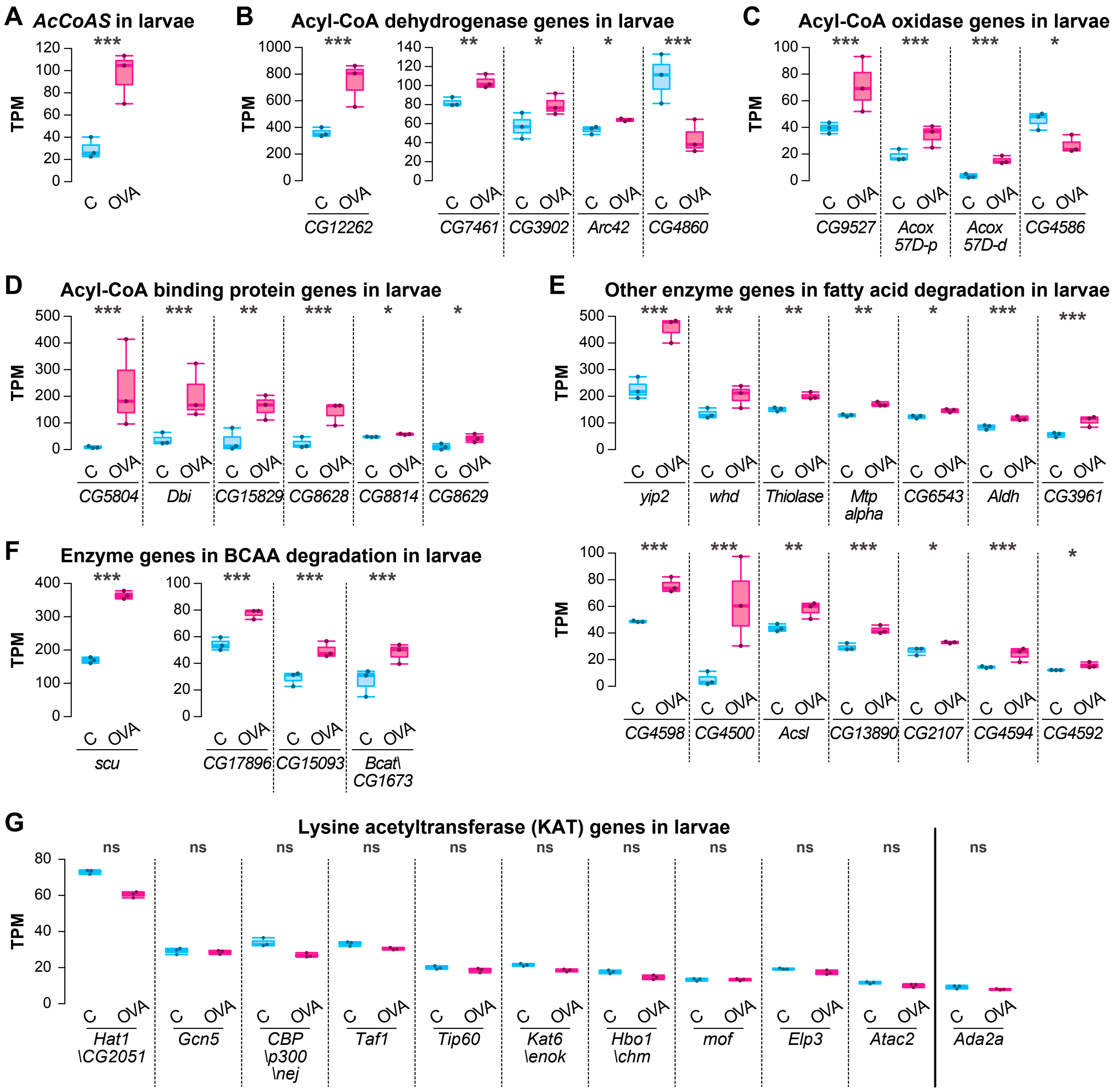
A AcCoAS in larvae
B Acyl-CoA dehydrogenase genes in larvae
C Acyl-CoA oxidase genes in larvae
D Acyl-CoA binding protein genes in larvae
E Other enzyme genes in fatty acid degradation in larvae
F Enzyme genes in BCAA degradation in larvae
G Lysine acetyltransferase (KAT) genes in larvae
TPM
C OVA
CG12262 CG7461 CG3902 Arc42 CG4860
CG9527 Acox 57D-p Acox 57D-d CG4586
CG5804 Dbi CG15829 CG8628 CG8814 CG8629
yip2 whd Thiolase Mtp alpha CG6543 Aldh CG3961
scu CG17896 CG15093 Bcat\CG1673
CG4598 CG4500 Acsl CG13890 CG2107 CG4594 CG4592
Hat1 \CG2051 Gcn5 CBP \p300 \nej Taf1 Tip60 Kat6 \enok Hbo1 \chm mof Elp3 Atac2 Ada2a

Appendix Figure S5. Gene expression in the OVA-fed male larvae, and the amount of pantothenate in the *nat3Δ*-fed male larvae or in the yeast strains

(A-G) Expression values (TPM) of genes in the control male larvae (sky blue, "C") that were fed on the control yeast diet (containing 0.05% Tween 80) or in the OVA-fed male larvae (magenta, "OVA") that were fed on the control yeast diet supplemented with oleic acid, valine, and acetic acid. (A) Acetyl-coenzyme A synthase (*AcCoAS*) gene. (B-E) 4 groups of the fatty acid degradation pathways: acyl-CoA dehydrogenase genes (B), acyl-CoA oxidase genes (C), acyl-CoA binding protein genes (D), and other genes including a 3-ketoacyl-CoA thiolase (acetyl-CoA acyltransferase) gene, *yip2*, and an acyl-CoA synthetase gene, *CG3961* (E). Most of the genes shown here were upregulated in the OVA-fed larvae, whereas *CG4860* in panel B and *CG4586* in panel C were downregulated. In each group, genes whose expression levels were not different (ns) between the control larvae and the OVA-fed larvae or not detected (undetected) are the following: *CG7461* (ns) and *CG9547* (ns) in group A; *CG5009* (ns) and *CG17544* (ns) in group B; *CG8498* (ns), *CG14232* (ns) and *CG33713* (undetected) in group C. (F) 4 differentially expressed genes of BCAA degradation pathways. The other differentially expressed BCAA degradation genes are in common with those in fatty acid degradation pathways. See also Figure EV3. (G) KAT genes. The OVA diet did not affect their expression. See other details in the legend for Appendix Figure S3D. Boxplots are depicted as in "Statistical analysis" in Methods. **P* < 0.05, ***P* < 0.01, ****P* < 0.001. The exact *P* values, sample sizes and statistical tests employed are listed in Dataset EV12.

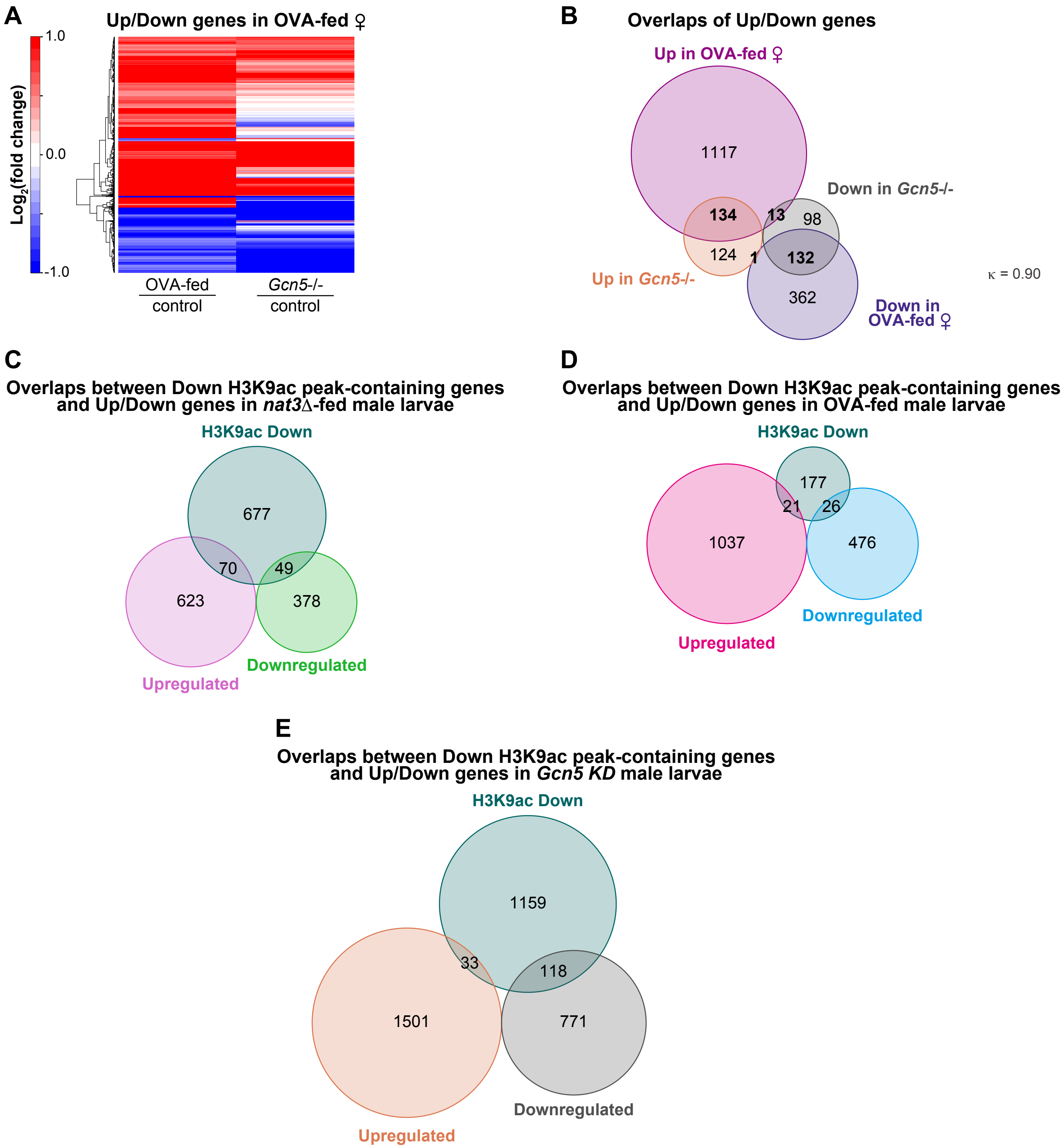
A
Up/Down genes in OVA-fed ♀
Log2(fold change)
1.0
0.0
-1.0
OVA-fed
control
Gcn5-/-
control
B
Overlaps of Up/Down genes
Up in OVA-fed ♀
1117
134
13
98
Down in Gcn5-/-
124
1
132
362
Up in Gcn5-/-
Down in OVA-fed ♀
κ = 0.90
C
Overlaps between Down H3K9ac peak-containing genes and Up/Down genes in nat3Δ-fed male larvae
H3K9ac Down
677
70
49
623
378
Upregulated
Downregulated
D
Overlaps between Down H3K9ac peak-containing genes and Up/Down genes in OVA-fed male larvae
H3K9ac Down
177
21
26
1037
476
Upregulated
Downregulated
E
Overlaps between Down H3K9ac peak-containing genes and Up/Down genes in Gcn5 KD male larvae
H3K9ac Down
1159
33
118
1501
771
Upregulated
Downregulated

Appendix Figure S6. A strong similarity of gene expression profiles between the OVA-fed female larvae and the *Gcn5* mutant larvae, and overlaps between Down peak-containing genes and Up/Down genes of the 3 male larvae groups

(A) A heat map showing whether each of the significantly upregulated (Up) and downregulated (Down) genes in the OVA-fed female larvae (red and blue, respectively, in "OVA-fed|control") tend to be up or down in the *Gcn5* mutant larvae (red or blue in "*Gcn5-/-*|control"). (B) Venn diagrams showing overlaps between the upregulated (Up) or downregulated (Down) genes in the OVA-fed female larvae and the Up or Down genes in the *Gcn5* mutant larvae. The numbers of genes in the individual categories are indicated. Changes in gene expression were highly correlated between the OVA-fed female larvae and the *Gcn5* mutant larvae. [κ_{Gcn5} = 0.90 (95% CI: 0.85-0.95); Cohen's kappa coefficient]. (C-E) Venn diagrams showing overlaps between genes that contain H3K9ac Down peaks and genes whose expression was upregulated or downregulated in the *nat3Δ*-fed male larvae (C), the OVA-fed male larvae (D) or the *Gcn5* knockdown male larvae (E). $^*P < 0.05$, $^{**}P < 0.01$, $^{***}P < 0.001$. The exact *P* values, sample sizes and statistical tests employed are listed in Dataset EV12.

References

Feller C, Forné I, Imhof A, Becker PB (2015) Global and specific responses of the histone acetylome to systematic perturbation. *Molecular Cell* 57: 559–571

Johnson et al. (2003) ohnson EC, Garczynski SF, Park D, Crim JW, Nässel DR, Taghert PH (2003) Identification and characterization of a G protein-coupled receptor for the neuropeptide proctolin in Drosophila melanogaster. *Proceedings of the National Academy of Sciences of the United States of America* 100: 6198–6203

Kounatidis I, Chtarbanova S, Cao Y, Hayne M, Jayanth D, Ganetzky B, Ligoxygakis P (2017) NF-κB Immunity in the Brain Determines Fly Lifespan in Healthy Aging and Age-Related Neurodegeneration. *Cell Reports* 19: 836–848

Niwa YS, Niwa R (2016) Ouija board: A transcription factor evolved for only one target in steroid hormone biosynthesis in the fruit fly Drosophila melanogaster. *Transcription* 7: 196–202

Pankotai T, Popescu C, Martín D, Grau B, Zsindely N, Bodai L, Tora L, Boros I (2010) Genes of the Ecdysone Biosynthesis Pathway Are Regulated by the dATAC Histone Acetyltransferase Complex in Drosophila. *Molecular and Cellular Biology* 30: 4254–4266

Thummel CS (1995) From embryogenesis to metamorphosis: The regulation and function of drosophila nuclear receptor superfamily members. *Cell* 83: 871–877
