# Supplementary material for: Growth phase diets diminish histone acetyltransferase Gcn5 function and shorten lifespan of Drosophila males
Source: EMBO Rep. 2025 Jul 10;26(15):3856–88. doi: 10.1038/s44319-025-00503-8 (PMC12332192; doi:10.1038/s44319-025-00503-8)
Supplement: Supplementary file 23 — Expanded View Figures [file 44319_2025_503_MOESM23_ESM.pdf]

Expanded View Figures

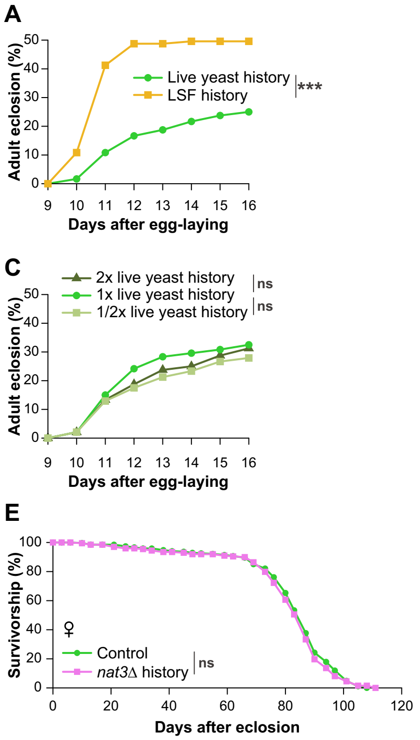

Figure EV1. Characterization of the live yeast–fly assay.

(**A**, **B**) Effects of the two distinct foods in larval stages on adult eclosion (**A**) and male lifespan (**B**): the modified synthetic complete medium (mSCM) plus live yeast (BY4741) in our live yeast–fly assay and LSF. (**A**) The numbers of emerging adults were much fewer on the live yeast diet ("Live yeast history") than on LSF ("LSF history"). In contrast to this major difference in the effect on the development, the difference in the lifespan was quite minor (**B**). These results indicate that our live yeast diet provides a less favorable environment for development compared to LSF, but the eclosed adults live essentially as long as the adults that developed on LSF from the very beginning of the larval stage. Adult eclosion percentage was calculated from the daily number of eclosed male and females. (**C**, **D**) Effects of amounts of live yeast (BY4741) on adult eclosion (**C**) and male lifespan (**D**). 40 µl of yeast suspensions with three different concentrations [1/2x, 1× (~8 × 10^8 cells/ml) and 2x] were added to each mSCM tube, and these tubes were cultured at 30 °C for 2 days before addition of germ-free fly embryos. Neither half nor double the quantity of live yeast produced a significantly different effect on larval development or on adult lifespan. These results suggest that differences in the growth of yeast single-gene KO strains on mSCM, if they are within the range tested here, do not affect development or lifespan. Adult eclosion percentage was calculated from the daily number of eclosed male and females. (**E**) The *nat3Δ* diet in larval stages did not affect female lifespan. *$P < 0.05$, **$P < 0.01$, ***$P < 0.001$. The exact *P* values sample sizes and statistical tests employed are listed in Dataset EV12. Source data are available online for this figure

© The Author(s)

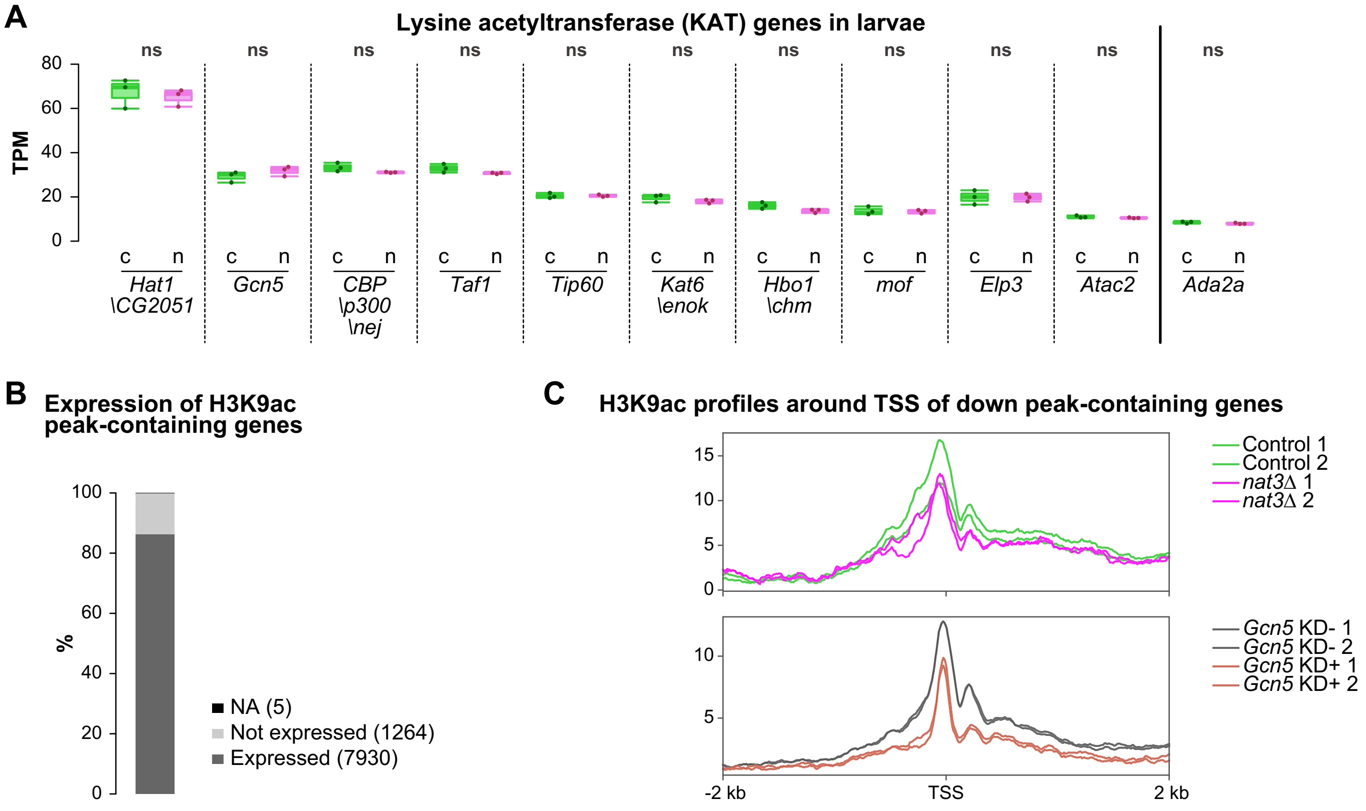

Figure EV2. Other characterizations of RNA-seq and CUT&RUN data related to the *nat3Δ*-fed male larvae and the *Gcn5* knockdown male larvae.

(**A**) Expression values (transcripts per million; TPM) of ATAC complex genes, including *Gcn5* and *Ada2a*, and other lysine acetyltransferase (KAT) genes in our larval whole-body RNA-seq data. None of the gene expressions examined were significantly different between the control and *nat3Δ* diets. We did this analysis because we assumed that feeding the *nat3Δ* diet partially diminished Gcn5 function in larvae on the basis of the data of Fig. 2B-2E, prompting us to ask whether any subunit genes of the ATAC complex were downregulated or not. The data of *Gcn5* and *Ada2a* (rightmost) are shown in the graph and that of other subunit genes (Torres-Zelada and Weake, 2021; Dent, 2024) are not shown. The other 9 genes in the graph encode *Drosophila* KATs, whose preferred target lysine residues in histones have been studied by mass spectrometry (Feller et al, 2015). (**B**) Breakdown of 9199 H3K9ac peak-containing genes of yeast-fed larvae. 86% are expressed in our RNA-seq data. 5 genes in "NA", *His3:CG33866*, *His3.3 A*, *His3.3B*, *His4r* and *DIP1*, are not included in our RNA-seq annotation gene list. (**C**) H3K9ac profiles around TSS of the Down peak-containing genes of the *nat3Δ*-fed larvae (top) and those of *Gcn5* KD+ larvae (bottom). The profiles of the individual H3K9ac replicates are shown. Boxplots are depicted as in "Statistical analysis" in "Methods". The exact *P* values, sample sizes and statistical tests employed are listed in Dataset EV12. Source data are available online for this figure

 © The Author(s)

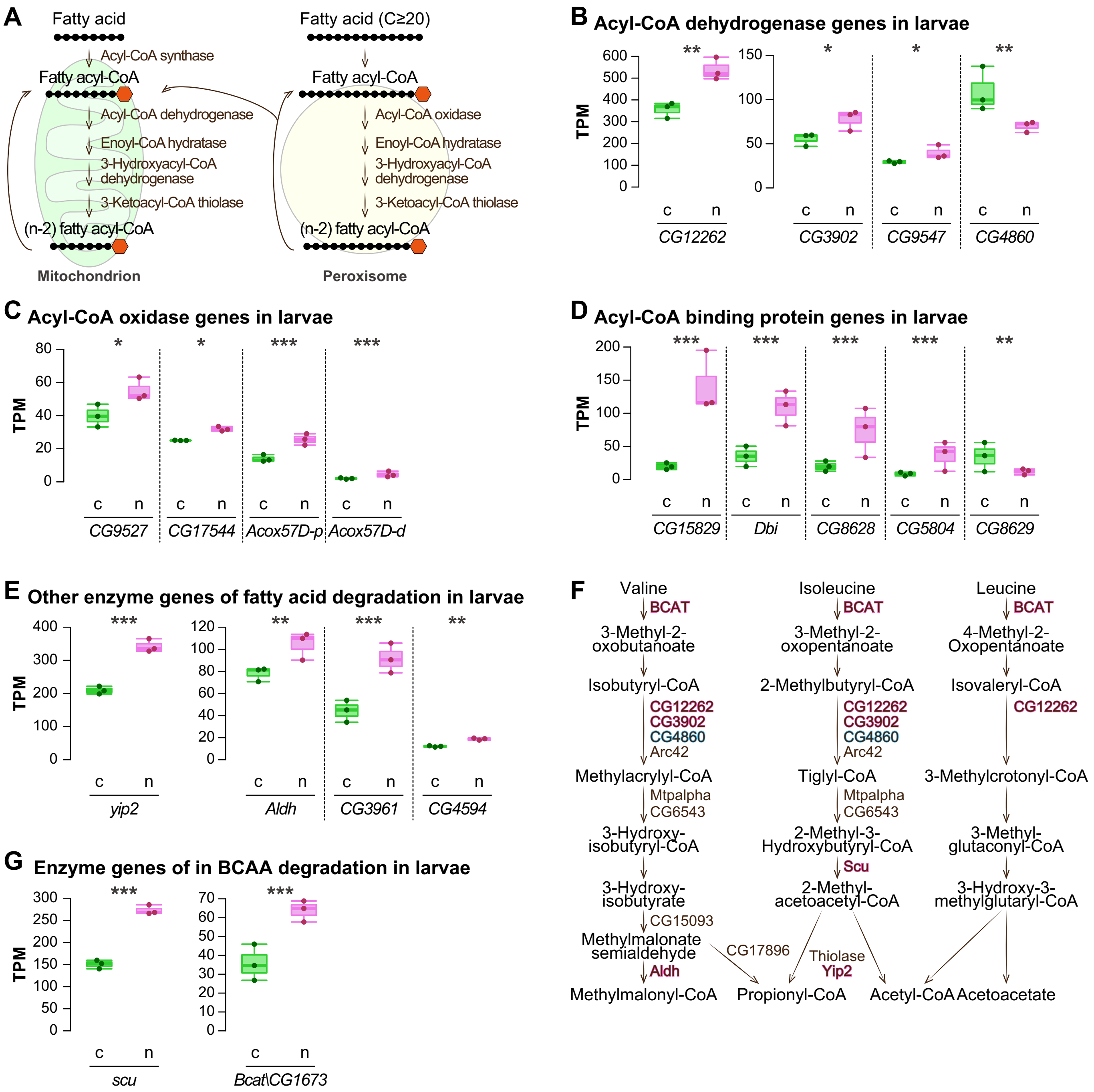
A
Fatty acid
Acyl-CoA synthase
Fatty acyl-CoA
Acyl-CoA dehydrogenase
Enoyl-CoA hydratase
3-Hydroxyacyl-CoA dehydrogenase
3-Ketoacyl-CoA thiolase
(n-2) fatty acyl-CoA
Mitochondrion
Fatty acid (C≥20)
Fatty acyl-CoA
Acyl-CoA oxidase
Enoyl-CoA hydratase
3-Hydroxyacyl-CoA dehydrogenase
3-Ketoacyl-CoA thiolase
(n-2) fatty acyl-CoA
Peroxisome
B Acyl-CoA dehydrogenase genes in larvae
TPM
CG12262 CG3902 CG9547 CG4860
C Acyl-CoA oxidase genes in larvae
TPM
CG9527 CG17544 Acox57D-p Acox57D-d
D Acyl-CoA binding protein genes in larvae
TPM
CG15829 Dbi CG8628 CG5804 CG8629
E Other enzyme genes of fatty acid degradation in larvae
TPM
yip2 Aldh CG3961 CG4594
G Enzyme genes of in BCAA degradation in larvae
TPM
scu Bcat\CG1673
F
Valine
BCAT
3-Methyl-2-oxobutanoate
Isobutyryl-CoA
CG12262
CG3902
CG4860
Arc42
Methylacrylyl-CoA
Mtpalpha
CG6543
3-Hydroxy-isobutyryl-CoA
3-Hydroxy-isobutyrate
CG15093
Methylmalonate semialdehyde
Aldh
Methylmalonyl-CoA
Isoleucine
BCAT
3-Methyl-2-oxopentanoate
2-Methylbutyryl-CoA
CG12262
CG3902
CG4860
Arc42
Tiglyl-CoA
Mtpalpha
CG6543
2-Methyl-3-Hydroxybutyryl-CoA
Scu
2-Methyl-acetoacetyl-CoA
CG17896
Thiolase
Yip2
Propionyl-CoA
Acetyl-CoA
Leucine
BCAT
4-Methyl-2-Oxopentanoate
Isovaleryl-CoA
CG12262
3-Methylcrotonyl-CoA
3-Methyl-glutaconyl-CoA
3-Hydroxy-3-methylglutaryl-CoA
Acetoacetate

Figure EV3. Elevated gene expression of the fatty acid degradation pathways and branched-chain amino acid (BCAA) degradation pathways in the *nat3Δ*-fed male larvae.

(**A**) β-Oxidation pathways of saturated fatty acids in mitochondria and peroxisomes, and representative enzymes that are conserved between *Drosophila* and mammals. (**B–E**) Gene expression values (TPM) of 4 groups of the fatty acid degradation pathways in our RNA-seq data from the control yeast-fed larvae (green, "c") and *nat3Δ*-fed larvae (pink, "n"): acyl-CoA dehydrogenase genes (**B**), acyl-CoA oxidase genes (**C**), long-chain fatty acyl-CoA binding protein genes (**D**), and other genes including a 3-ketoacyl-CoA thiolase (acetyl-CoA acyltransferase) gene, *yip2*, and an acyl-CoA synthetase gene, *CG3961* (**E**). Most of the genes shown here were upregulated in the *nat3Δ*-fed larvae, whereas *CG4860* in (**B**) and *CG8629* in (**D**) were downregulated. In each group, genes whose expression levels were not different (ns) between the control yeast-fed larvae and the *nat3Δ*-fed larvae or not detected (undetected) are the following: *CG7461* (ns) and *Arc42* (ns) in group B; *CG5009* (ns) and *CG4586* (ns) in group C; *CG8498* (ns), *CG8814* (ns), *CG14232* (ns) and *CG33713* (undetected) in group D. (**F**) BCAA degradation pathways in mitochondria of *D. melanogaster* that are partially shared by β-Oxidation pathways. Differentially expressed enzyme genes are shown as below: those shaded in magenta are upregulated in *nat3Δ*-fed larvae and/or in the OVA-fed larvae; CG4860 in sky blue is downregulated in both the *nat3Δ*-fed larvae and the OVA-fed larvae, and the others are upregulated only in the OVA-fed larvae (panel B; Appendix Fig. S5B,E,F). (**G**) Expression values (TPM) of 2 differential genes of BCAA degradation pathways in the control yeast-fed larvae (green, "c") and the *nat3Δ*-fed larvae (pink, "n"). The other differential BCAA degradation genes are common with those in fatty acid degradation pathways. See panels (**B**, **E**). Boxplots are depicted as in "Statistical analysis" in "Methods". *$P < 0.05$, **$P < 0.01$, ***$P < 0.001$. The exact *P* values, sample sizes and statistical tests employed are listed in Dataset EV12. Source data are available online for this figure

© The Author(s)

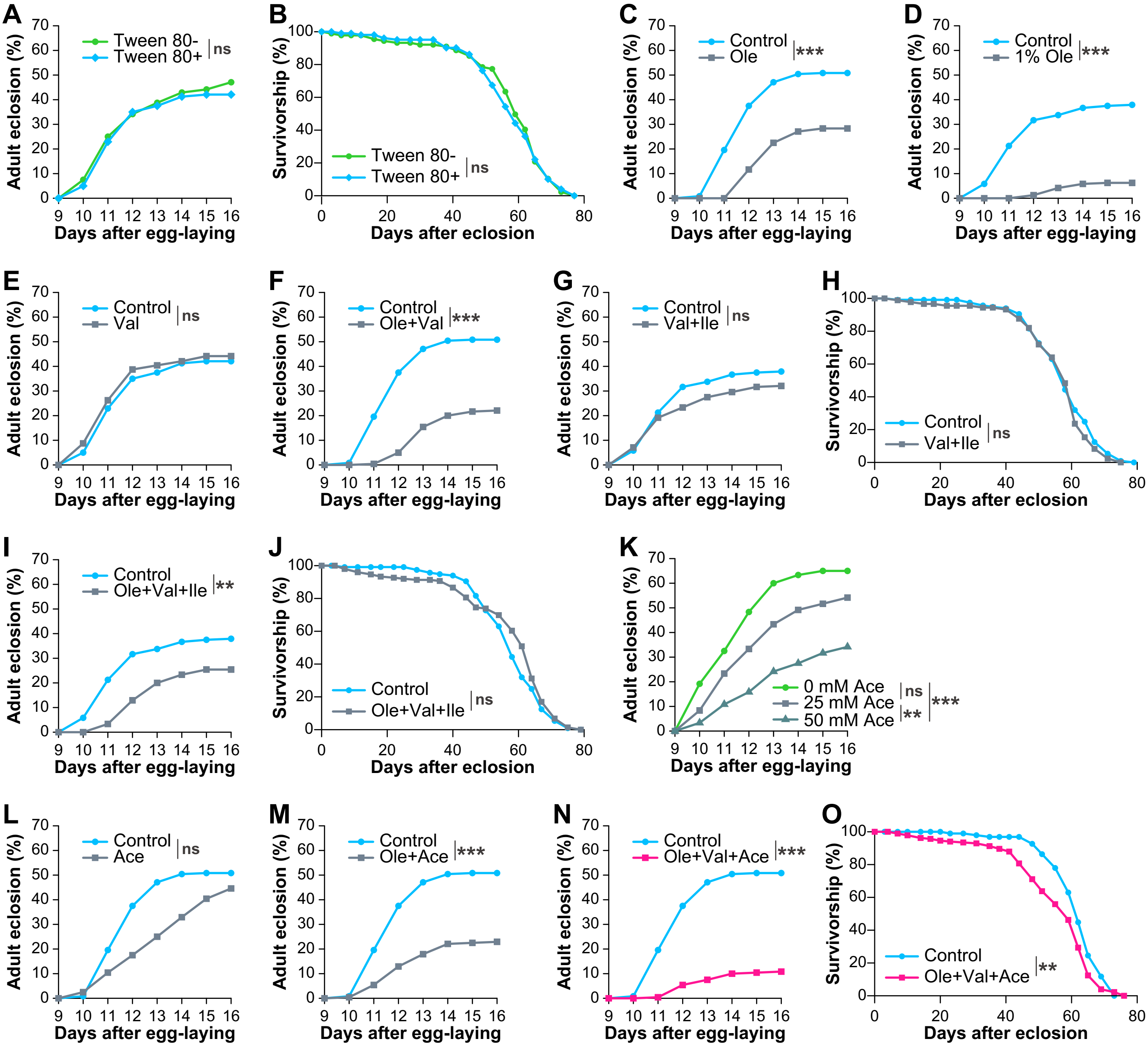

Figure EV4. Effects of supplemented reagents on adult eclosion and male lifespan.

(**A**, **B**) Addition of 0.05% Tween 80 to the control yeast diet for larvae did not affect adult eclosion (**A**) nor male lifespan (**B**). Adult eclosion percentage was calculated from the daily number of eclosed male and females. (**C–O**) Effects of supplemented reagents on adult eclosion (**C–G**, **I**, **K–N**) and male lifespan (**H**, **J**, **O**). Throughout this figure, control larvae ("Control") were fed on the live control yeast and the agar medium (mSCM) containing Tween 80 and developed to adults (sky blue). The data of the experimental groups are colored in gray. Adult eclosion percentage was calculated from the daily number of eclosed adult flies of both the sexes. Abbreviations: Ole (oleic acid), Val (valine), Ile (isoleucine), Ace (acetic acid). *$P < 0.05$, **$P < 0.01$, ***$P < 0.001$. The exact P values, sample sizes and statistical tests employed are listed in Dataset EV12. Source data are available online for this figure

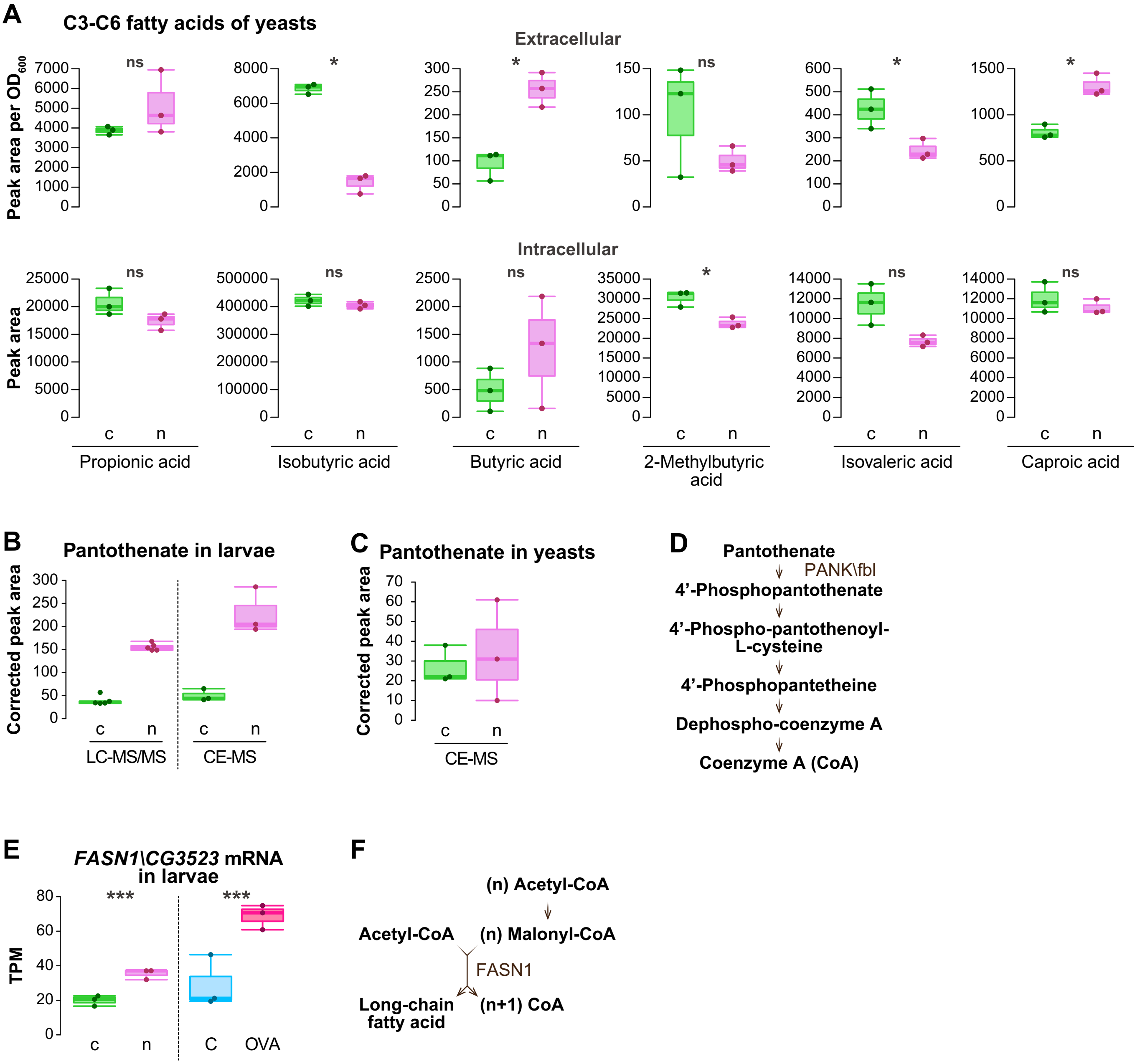

Figure EV5. Abundance of short-chain fatty acids and pantothenate of the yeast strains and/or the yeast-fed male larvae, and expression of *Fatty acid synthase1* (*FASN1*) in the larvae.

(**A**) Relative amounts of short-chain (C3-C6) fatty acids in the liquid culture media ("Extracellular") and in the yeast cells ("Intracellular") of the control or *nat3Δ* yeast strain ("c" or "n"). The vertical axes show corrected peak areas by OD_{600} values or raw values from liquid chromatography-mass spectrometry (LC-MS). (**B**–**D**) Relative amounts of pantothenate in the yeast-fed larvae (**B**) and in the yeast strains (**C**). The vertical axes show peak areas that were measured in metabolome analyses (LC-MS/MS or CE-MS) and corrected by individual sample weights. Samples of either the control yeast-fed larvae or the control yeast are shown in the "c" boxplots (green), while samples of the *nat3Δ*-fed larvae or the *nat3Δ* yeast are shown in the "n" boxplots (pink). Logarithmic transformation and subsequent *t* test of MetaboAnalyst showed that pantothenate was significantly more abundant in the *nat3Δ*-fed larvae (left: $P < 0.001$, $FDR < 0.001$, right: $P < 0.01$, $FDR = 0.064922$). On the other hand, the pantothenate amount was not significantly different between the control and *nat3Δ* yeasts ($P = 0.967$). See more details in Datasets EV3 and EV5. (**D**) The biosynthetic pathway for CoA production. Pantothenate is first phosphorylated by pantothenate kinase (PANK). PANK is a rate-limiting enzyme in this pathway and feedback regulated by the end product, CoA, and also by acetyl-CoA and acyl-CoA species. (**E**) Expression values (TPM) of *FASN1\CG3523* in the *nat3Δ*-fed larvae (pink, "n"), the OVA-fed larvae (magenta, "OVA") and the respective controls (green, "c"; sky blue "C"). (**F**) Generalized enzymatic reaction of FASN1: a long-chain fatty acid and CoAs are produced from acetyl-CoAs in the cytoplasm. Boxplots are depicted as in "Statistical analysis" in Methods. $^*P < 0.05$, $^{**}P < 0.01$, $^{***}P < 0.001$. The exact *P* values, sample sizes and statistical tests employed are listed in Dataset EV12. Source data are available online for this figure

 © The Author(s)

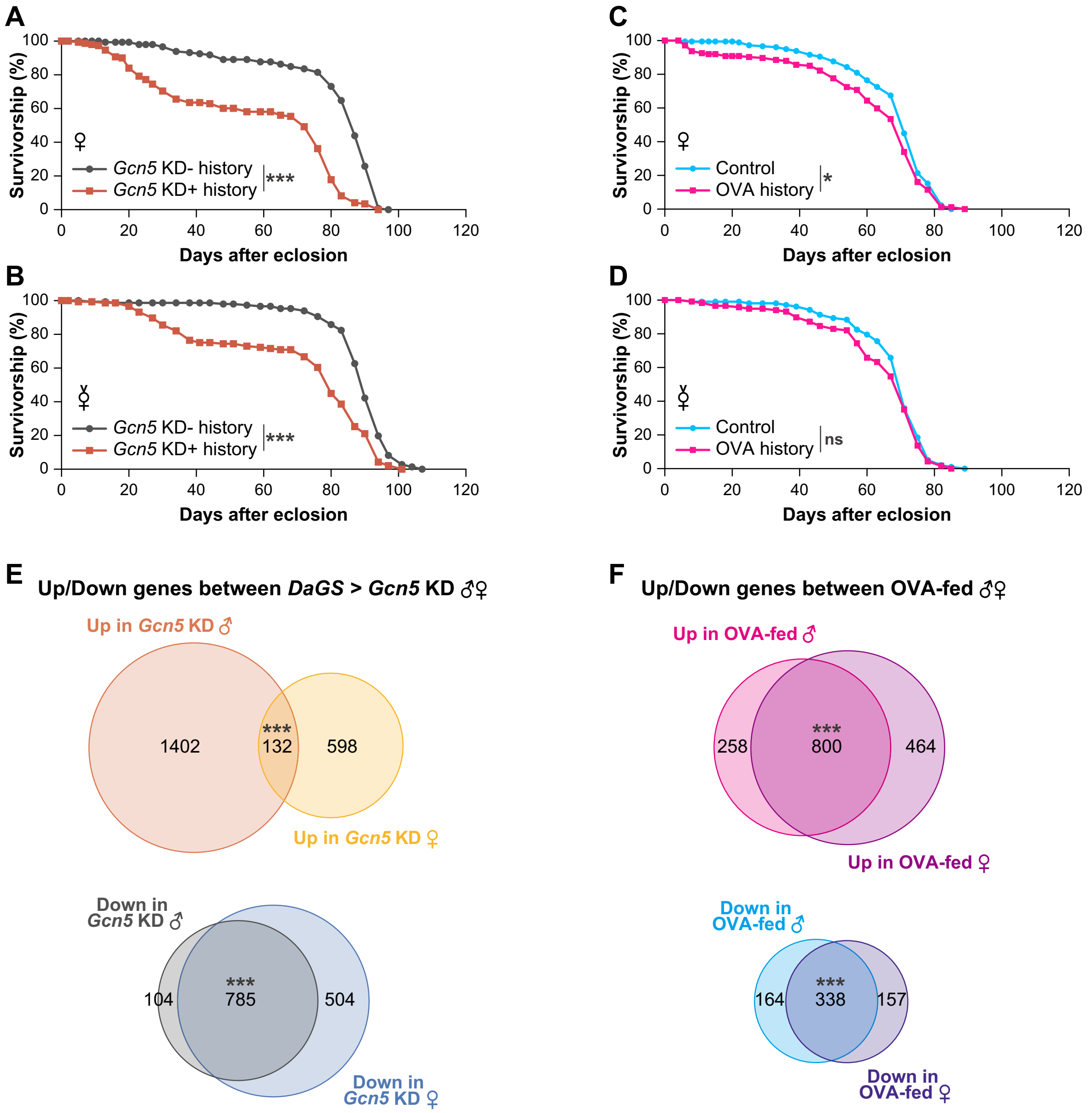

Figure EV6. Impacts of the OVA diet or *Gcn5* knockdown in larval stages on gene expression of female larvae and female lifespan.

(**A**, **B**) Survival curves of mated females (**A**) and virgin females (**B**) with or without the *Gcn5* KD history in larval stages. (**C**, **D**) Survival curves of mated females (**C**) and virgin females (**D**) with or without the OVA nutrition history in larval stages. (**E**, **F**) Comparisons of gene expression between male larvae and female larvae under the *Gcn5* KD condition (**E**) or the OVA diet condition (**F**). Venn diagrams showing overlaps of Up or Down genes between the sexes. The overlaps were significant in the individual comparisons and particularly large regarding the Down genes in the *Gcn5* KD condition, and both the Up and the Down genes in the OVA diet condition. All these data were obtained in a set of experiments. $^{*}P < 0.05$, $^{**}P < 0.01$, $^{***}P < 0.001$. The exact *P* values, sample sizes and statistical tests employed are listed in Dataset EV12. Source data are available online for this figure
